# Hemolymph Metabolism Analysis of Honey Bee (*Apis mellifera* L.) Response to Different Bee Pollens

**DOI:** 10.3390/insects14010037

**Published:** 2022-12-30

**Authors:** Hongcai Chang, Guiling Ding, Guangqun Jia, Mao Feng, Jiaxing Huang

**Affiliations:** 1 Key Laboratory for Insect-Pollinator Biology of the Ministry of Agriculture and Rural Affairs, Institute of Apicultural Research, Chinese Academy of Agricultural Sciences, Beijing 100093, China; 2 Technology Center of Qinhuangdao Customs, Qinhuangdao 066004, China

**Keywords:** bee pollen, amino acid, *Apis mellifera* L., hemolymph, metabolomic

## Abstract

**Simple Summary:**

Honey bees collect pollen with different nutrients to meet their physiological metabolism needs and maintain colony development. The protein contents vary among bee pollens, and the nutritional level of pollen is mainly reflected in the amino acid composition of its proteins. In northern China, pear, rape, and apricot plants are monoculture plants grown in a large area, and their pollen is collected by honey bee colonies during the spring. However, honey bees showed a foraging preference for apricot or rape pollen. It is important to understand the differences in the amino acid levels of these three pollen species and the associated physiological metabolic responses in honey bees. In this study, we evaluated the differences in amino acid content and hemolymph metabolism in caged adult bees that were fed three different bee pollen types. The numbers and levels of essential amino acids in pear pollen were significantly higher than those in apricot and rape pollen. Furthermore, these three bee pollens induced different metabolic responses in adult honey bees of different ages. Overall, pear pollen had the highest nutritional value among the three bee pollens in terms of amino acid level, and bee pollen was shown to play an important role in the pathways related to amino acid and lipid metabolism in bees in early adulthood.

**Abstract:**

Pollen is essential to the development of honey bees. The nutrients in bee pollen vary greatly among plant species. Here, we analyzed the differences in the amino acid compositions of pear (*Pyrus bretschneideri*), rape (*Brassica napus*), and apricot (*Armeniaca sibirica*) pollens and investigated the variation in hemolymph metabolites and metabolic pathways through untargeted metabolomics in caged adult bees at days 7 and 14. The results showed that the levels of five essential amino acids (isoleucine, phenylalanine, lysine, methionine, and histidine) were the highest in pear pollen, and the levels of four amino acids (isoleucine: 50.75 ± 1.93 mg/kg, phenylalanine: 87.25 ± 2.66 mg/kg, methionine: 16.00 ± 0.71 mg/kg and histidine: 647.50 ± 24.80 mg/kg) were significantly higher in pear pollen than in the other two kinds of bee pollen (*p* < 0.05). The number of metabolites in bee hemolymph on day 14 (615) was significantly lower than that on day 7 (1466). The key metabolic pathways of bees, namely, “sphingolipid metabolism (*p* = 0.0091)”, “tryptophan metabolism (*p* = 0.0245)”, and “cysteine and methionine metabolism (*p* = 0.0277)”, were significantly affected on day 7. There was no meaningful pathway enrichment on day 14. In conclusion, pear pollen had higher nutritional value among the three bee pollens in terms of amino acid level, followed by rape and apricot pollen, and the difference in amino acid composition among bee pollens was reflected in the lipid and amino acid metabolism pathways of early adult honey bee hemolymph. This study provides new insights into the physiological and metabolic functions of different bee pollens in bees.

## 1. Introduction

The growth and development of honey bee colonies require sufficient nutrients, the most essential among which are sugars, proteins, and lipids contained in nectar and pollen to meet their nutritional metabolism needs. Pollen provides almost all the proteins required by the colony and plays an important role in the growth and development of bees [1,2]. The protein content of pollen differs among plant species, resulting in significant variability in the amino acid contents and physiological metabolism [3,4,5]. Currently, the cultivation of single crops on a large scale can lead to pollen scarcity in certain periods or to the presence of low-protein pollen for honey bees [6]. Pollen proteins differ from each other, which has a great impact on the survival and growth of honey bees. It is necessary to further understand the differences in pollen protein composition and metabolic components.

Pollen contains nutrients such as protein, lipids, carbohydrates, starch, sterols, vitamins, and minerals, all of which are essential nutrients for honey bees [7,8]. However, the nutritional value is usually evaluated based on its protein content [9]. High-protein pollen is generally more beneficial to colony growth and development than low-protein pollen [10,11]. In addition, the nutritional quality of pollen decreases if the essential amino acids required by honey bees are insufficient in high-protein pollen [11]. Early studies showed that methionine, arginine, tryptophan, lysine, isoleucine, phenylalanine, histidine, valine, leucine, and threonine were essential amino acids for honey bees, while tyrosine, cysteine, serine, hydroxyproline, alanine, glycine, and proline were nonessential [12]. Of the essential amino acids, leucine, isoleucine, and valine were required in the greatest amounts (most essential amino acids); tryptophan, methionine, and histidine in the lowest amounts (least essential amino acids); and threonine, phenylalanine, arginine, and lysine in intermediate amounts [9]. Bee pollen from different plant species differs in amino acid composition and concentration. Pollens with a high proportion of essential amino acids are generally considered assumed to be of greater nutritional value than those with a low proportion of essential amino acids [7]. Therefore, a comparison of the amino acid composition of different bee pollens can provide a powerful basis for evaluating the nutritional differences among bee pollens [7,9].

Pear (*Pyrus bretschneideri*), rape (*Brassica napus*), and apricot (*Armeniaca sibirica*) are monoculture plants grown in a large area in China and their pollen is collected by honey bee colonies in spring [13]. The blooming periods of these plants are relatively concentrated and overlapping, and honey bees may selectively collect more pollen from certain plants. This causes the colony to feed on fewer types of pollen. The foraging preferences of adult bees are also affected by pollen amino acid composition [9]. In addition, pear flowers were shown to be not as attractive to bees as rape flowers, possibly due to the low volume and sugar concentration of pear nectar or the significant impact of pear volatiles [14,15,16]. Caged honey bees showed a significant preference for apricot pollen over pear pollen [17]. Honey bee foraging preferences appeared to reflect differences in amino acid composition among pollens [9]. It is essential to explore the difference in amino acid composition among the three kinds of bee pollen.

The pollen consumption behavior of bees varies with age. Pollen is consumed primarily during the first 3 to 5 days of an adult worker’s life [18]. Honey bees are considered adults after emergence, but substantial growth occurs during the first 6 days [12]. Nutritional quality assessment by honey bees may occur directly through pollen nutrient perception or indirectly through physiological or larval feedback [19]. Adult *Apis mellifera* bees can sense the pollen’s nutritional composition via taste [20,21]. However, the physiological processes associated with different pollen nutrients in bees are poorly understood.

Metabolomics is an analytical tool that can be used to quickly and quantitatively measure the changes in a range of metabolites in response to an external stressor and has been shown to have some advantages over other postgenomic technologies [22]. Unlike the transcriptome or the proteome, which vary from species to species, the basic metabolic pathways and their metabolites are similar among different species, making metabolomic analysis much more universally applicable [23]. Metabolomics approaches have been successfully applied to biomarker discovery, for risk assessment of toxic exposures, and to examine the metabolic responses to environmental stressors [24]. Metabolomic studies have shown that gluconeogenesis contributes significantly to blood sugar stability in bumble bees maintained on a low-carbohydrate diet [25]. In addition, protein-containing nutrition in adult worker honey bees can trigger certain metabolic responses [26]. Different pollen nutrients play an important role in bee immunity, and a low-protein diet can reduce the expression of host-specific immunity-related genes in bumble bees [26,27]. It is crucial to study the metabolic responses to pollens with differences in nutritional value in adult bees. Hemolymph is the only biofluid circulating in the honey bee body and is critical for metabolite biomarker discovery [25]. Metabolomic methods can be suitably applied to metabolism-related research on honey bees feeding on different pollens.

In this study, we evaluated the differences in the amino acid composition of three bee pollens: pear (*P. bretschneideri*), rape (*B. napus*), and apricot (*A. sibirica*) pollens. We also investigated changes in hemolymph metabolites and metabolic pathways in caged adult bees fed the three types of pollen for 7 and 14 days using untargeted metabolomics to understand whether feeding pollen at different ages has different physiological effects on bees. The following questions were investigated: (1) What are the differences in the amino acid composition of these three bee pollens? (2) What are the differences among these three bee pollens in adult workers at the metabolic level? (3) What are the differences in bee pollen metabolism among bees of different ages?

## 2. Materials and Methods

### 2.1. Pollen and Amino Acid Analysis

Three single bee pollen types (pear, rape, and apricot) were fed to adult bees. Bee pollen was collected from Western honey bee colonies with pollen traps in the spring of 2020. Pear pollen was collected in a large-scale planting orchard in Yuncheng, Shanxi Province, China. Rape and apricot pollen were purchased from a commercial company (Beijing China-Bee Science & Technology Development Co., Ltd., Beijing, China.). The bee pollens were examined for purity under a microscope with 10 replicates and more than 100 bee pollen grains per replicate. The bee pollen purity was confirmed to be more than 99%. All fresh bee pollen samples were stored at −80 °C until use.

A total of 0.5 g of bee pollen was weighed and suspended in 10 mL of 0.1 mol/L hydrochloric acid. The suspension was ultrasonicated using a water bath and mixed in a vortex until the sample dissolved completely. One milliliter of the pollen suspension was transferred to a 10 mL plug test tube. The internal standard n-leucine solution (40 μL), phenyl isothiocyanate-acetonitrile solution (1:1) (500 μL), triethylamine-acetonitrile solution (1:2) (500 μL), and 100 μL acetic acid solution (1:3) were added. Two milliliters of n-hexane was added and the sample was vortexed for 1 min. The upper suspension was discarded after an obvious separate layer formed. The sample was filtered with a 0.45 μM organic filter membrane (Agilent Technologies, Palo Alto, CA, USA) into a 1.5 mL vial and the volume was brought up to 1 mL prior to Liquid Chromatograph Mass Spectrometer (LC-MS) (Quattro Premier XE, Waters Corp., Milford, MA, USA) analysis.

The system used a UPLC BEH C18 column (2.1 mm × 100 mm, 1.7 μm). Mobile phases A and B were acetonitrile and 5% acetonitrile (containing 10 mmol/L ammonium acetate), respectively. The gradient was as follows: 0–1 min, 99% B; 1–2.5 min, 82% B; 2.5–5.5 min, 82% B; 30 min, 90% B; 5.5–6.5 min, 20% B; 6.5–7.5 min, 20% B; 7.5–8 min, 99% B; 8–12 min, 99% B. The flow rate was 300 μL/min, the injection volume was 2 μL, and the column temperature was 40 °C. Positive ionization mode was used. The mass spectrometry parameters were set as follows: capillary voltage, 3.0 kV; ion source temperature, 120 °C; lens voltage, 0.1 V; desolvation temperature, 350 °C; desolvation gas (N_2_) flow rate, 650 L/h; carrier gas (N_2_) flow rate, 50 L/h; collision gas (Ar) flow rate, 0.15 mL/min. The purity of amino acid standard is greater than or equal to 95% (Sigma-Aldrich, Shanghai, China). Amino acid content was measured according to the external standard quantitative method. The calibration curves were established, with a correlation coefficient (r) higher than 0.99. The limit of detection (LOD) and the limit of quantitation (LOQ) for 20 amino acid derivatives were less than 0.1 mg/kg and 0.3 mg/kg, respectively. Recoveries were evaluated by adding the standards into the bee pollen sample at two levels, varying from 80 to 136% and the relative standard deviation (RSD) of precision was in the range of 4.3–34.6%.

### 2.2. Honey Bee Feeding and Hemolymph Collection

This study was conducted from June to August 2020 at the Institute of Apicultural Research, Chinese Academy of Agricultural Sciences (IAR-CAAS). Frames of emerging broods collected from five queenright *A. mellifera* colonies were placed in an incubator (Shanghai Yiheng Technology Co., Ltd., Shanghai China) at 35 °C and 50% relative humidity. Eighty newly emerged workers were collected in wood cages with wire mesh (13 × 14 × 14 cm). The queenless workers were kept at 30 °C and 50% relative humidity. Fifteen cages were set up with 5 cages per pollen group. The workers were fed a 50% (*w*/*w*) sucrose solution and supplied with one of three types of pollen pellets mixed with sucrose solution. The pollen pellets and sucrose solution were supplied and changed in ELISA plate strips (Solarbio Biotechnology Co., Ltd., Beijing, China) every 24 h to ensure freshness.

Bee hemolymph was collected by detaching an antenna, followed by the application of delicate pressure to the bee’s abdomen and extraction using Wiretrol II capillary micropipettes (VWR) [28]. Hemolymph samples were collected on days 7 and 14 after feeding with different pollens. Each hemolymph sample was collected in centrifuge tubes (Axygen, Union City, CA, USA), immediately placed on ice, and stored at −80 °C until chemical analysis. Hemolymph collection was performed under a binocular microscope (Olympus SZX17, Olymplus Corporation, Tokyo, Japan), and three rules were strictly followed to guarantee the quality of sampling: (i) the hemolymph was pure and transparent; (ii) no other tissues were perforated; and (iii) the sampling time (incision and extraction) per bee was less than 35 s.

### 2.3. Extraction of Polar Metabolites from Bee Hemolymph

We used methanol as the solvent to extract metabolites from honey bee hemolymph. Forty microliters of hemolymph sample were mixed with 160 μL of methanol to remove proteins [25]. Samples were incubated at 4 °C for 1 h to enhance protein precipitation and centrifuged at 15,000× *g* for 5 min at 4 °C to remove the precipitate. The supernatant was transferred to an LC-MS vial with a glass insert. The quality control (QC) sample, which was a mixture of equal masses of each sample, was used to assess the variance in data.

### 2.4. UPLC-QTOF-MS Analysis

Chromatographic separation of the hemolymph extract was conducted on an Agilent 1290 Infinity II UPLC system (Agilent Technologies, Palo Alto, CA, USA). The system was equipped with an Acquity UPLC BEH C18 column (2.1 mm × 100 mm, 1.7 μm; Waters Corp., Milford, MA, USA). Mobile phases A and B were 95% ACN (containing 10 mM ammonium formate and 1 μL of formic acid) and 50% ACN (containing 10 mM ammonium formate and 1 μL of formic acid), respectively. In negative ionization mode, mobile phases A and B were 95% ACN (containing 10 mM ammonium acetate and adjusting the pH to 9 with ammonium hydroxide solution) and 50% ACN (containing 10 mM ammonium acetate, pH 9.0), respectively. In positive ionization mode, the solvent gradient elution was set as follows: 0−2 min, 1% B; 2–3.5 min, 1–20% B; 3.5–17 min, 20–80% B; 17–17.5 min, 80–99% B; 17.5–19 min, 99% B; 19–19.1 min, 99–1% B; and 19.1–22 min, 1% B. The following program was used in negative ionization mode: 0−2 min, 5% B; 2−4 min, 5−20% B; 4–18 min, 20–85% B; 18–19 min, 85–95% B; 19–21 min, 95% B; 21–21.1 min, 95–5% B; and 21.1–25 min, 5% B. The injection volume was 1 μL, and the flow rate was set to 300 μL/min.

The UPLC system was connected to an Agilent 6510 ESI-Q-TOF high-resolution accurate-mass spectrometer (Agilent Technologies, Palo Alto, CA, USA). Positive ionization mode was used. The mass spectrometry parameters were set as follows: capillary voltage, 4.0 kV; fragmentor voltage, 135 V; skimmer voltage, 65 V; collision energy, 40 eV; gas temperature, 350 °C; drying gas flow rate, 11 L/min; nebulizer pressure, 40 psi; and *m*/*z* range, 100−1500. Nitrogen was utilized for drying, nebulization, and collision. Agilent Mass Hunter workstation software (Agilent Technologies, Palo Alto, CA, USA) was applied for data analysis and compound identification.

### 2.5. Data Analysis

All amino acid content statistics were examined with SPSS 26.0 (IBM, Armonk, NY, USA) and are presented as the mean ± standard error. The data were first checked for normality (Kol-mogorov–Smirnov and Shapiro–Wilk tests). When the data fit a normal distribution, the amino acid levels in the three pollen types were examined using one-way ANOVA and least significant difference (LSD) multiple comparisons. The means were judged to be substantially different when the *p* value was less than 0.05.

Untargeted metabolomic data were collected in positive ion mode. The dataset was analyzed on the Mass Profiler Professional (MPP) (Agilent Technologies, Palo Alto, CA, USA) to generate a data matrix consisting of retention time, mass-to-charge ratio, and peak intensity. Metabolites were identified on the basis of the METLIN database and screened with a score greater than 80 and mass error less than 5 ppm (i.e., 0.0005%) [29]. Principal component analysis (PCA) was performed to preliminarily understand the relationships among the data matrixes. To further determine the differences in metabolite composition among samples from the same group, supervised signal correction was applied to a partial least square-discriminant analysis (PLS-DA) model, and orthogonal partial least square-discriminant analysis (OPLS-DA) was used to identify the differences between different groups. To identify the metabolic pathways affected by different pollens, the OPLS-DA model compared the metabolite content of the samples in pairs to screen the differentially abundant metabolites, and a detailed map of related pathways was constructed using the reference map deposited in the KEGG database. Based on the OPLS-DA model, variable importance in projection (VIP) values were obtained, and the metabolites were considered significantly differentially abundant if the *p* value was <0.05 (Student’s *t* test), VIP value was >1.0, and fold change (FC) was >2 or <0.5. The pathways associated with the differentially abundant metabolites were identified using MetaboAnalyst 5.0 (https://www.metaboanalyst.ca/, accessed on 8 September 2022) and KEGG (https://www.genome.jp/kegg/, accessed on 9 September 2022).

## 3. Results

### 3.1. Amino Acid Components of Pollen

**Figure 1:**
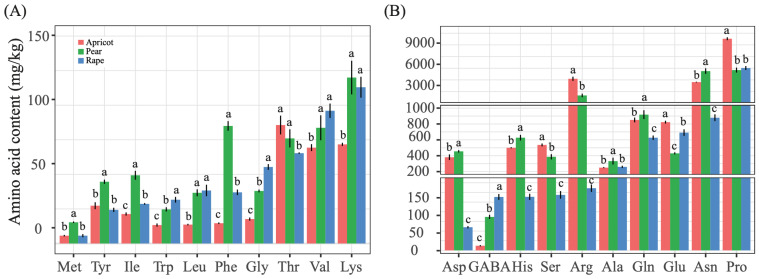
Levels of different amino acids in pear (*Pyrus bretschneideri*), rape (*Brassica napus*) and apricot (*Armeniaca sibirica*) pollen. (**A**) Met = methionine, Tyr = tyrosine, Ile = isoleucine, Trp = tryptophan, Leu = leucine, Phe = phenylalanine, Gly = glycine, Thr = threonine, Val = valine, and Lys = lysine. (**B**) Asp = aspartic acid, GABA = γ-aminobutyric acid, His = histidine, Ser = serine, Arg = arginine, Ala = alanine, Gln = glutamine, Glu = glutamic acid, Asn = asparagine, and Pro = proline. Different letters indicate a significant difference (*p* < 0.05).

A total of 19 amino acids and 1 nonprotein amino acid (γ-aminobutyric acid, GABA) were found in pear, rape, and apricot pollens (Figure 1). Investigation of the amino acid composition of the three pollens revealed significant differences in amino acids among the three bee pollens. Among the most essential amino acids, the levels of leucine and valine in pear and rape pollens were significantly higher than those in apricot pollen (*p* < 0.05), and isoleucine had the highest level in pear pollen (50.75 ± 1.93 mg/kg). Among the amino acids of intermediate importance, the levels of threonine and arginine in pear and apricot pollens were significantly higher than those in rape pollen (*p* < 0.05), the level of isoleucine in pear and rape pollens differed significantly from that in apricot pollen, and phenylalanine had the highest level in pear pollen (87.25 ± 2.66 mg/kg). Furthermore, among the least essential amino acids, the levels of methionine (16.00 ± 0.70 mg/kg) and histidine (647.50 ± 24.80 mg/kg) in pear pollen were significantly higher than those in the other two pollens. Tryptophan had the highest level in rape pollen (31.67 ± 1.20 mg/kg). Proline was the most abundant amino acid in the three pollens, and its level in apricot pollen (9777.50 ± 78.11 mg/kg) was significantly higher than that in the other two pollens (rape: 6028.33 ± 146.45, pear: 5877.50 ± 174.04) (*p* < 0.05) (Figure 1).

### 3.2. Metabolite Data Modeling

**Figure 2:**
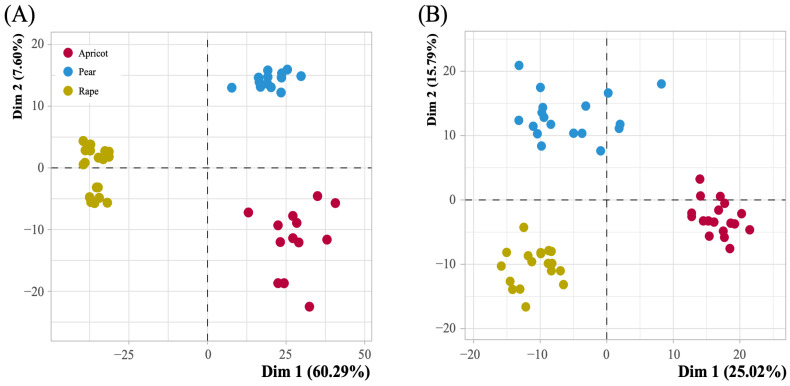
PCA score plots from multivariate statistical analysis. (**A**) Score plots for hemolymph metabolites after 7 days of bee pollen feeding (number of apricot, rape, and pear samples: 13, 17, and 13, respectively). (**B**) Score plots for hemolymph metabolites after 14 days of pollen feeding (number of apricot, rape, and pear samples: 17, 18, and 17, respectively).

**Figure 3:**
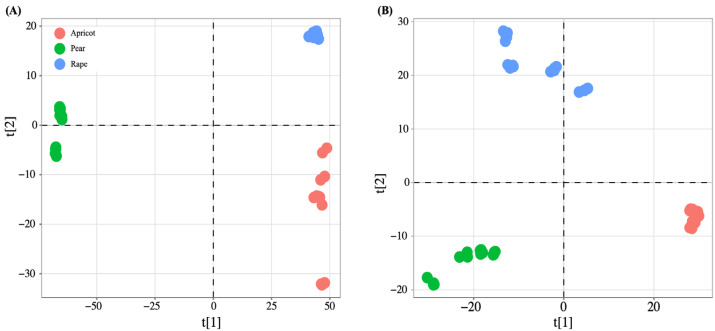
PLS-DA score plots from multivariate statistical analysis, t[1] and t[2] represent the first and second predictive principal components, respectively. (**A**) Score plots for hemolymph metabolites after 7 days of bee pollen feeding (number of apricot, rape, and pear samples: 13, 17, and 13, respectively). (**B**) Score plots for hemolymph metabolites after 14 days of pollen feeding (number of apricot, rape, and pear samples: 17, 18, and 17, respectively).

Compared to negative ion mode, in positive ion mode, 1466 and 615 components were obtained from the day 7 and day 14 hemolymph samples, respectively. PCA distinguished the hemolymph metabolites of three pollen-fed caged worker bees on day 7 and day 14. The PCA results showed a total of 67.89% and 40.81% model variance described by the PC1 and PC2 axes on day 7 and day 14, respectively (Figure 2). The PLS-DA model compared the different pollen metabolite levels of the samples in pairs to evaluate the difference (day 7: R2X = 0.958, R2Y = 0.999, Q2 = 0.999; day 14: R2X = 0.664, R2Y = 0.998, Q2 = 0.997) (Figure 3).

### 3.3. Metabolic Pathway Analysis

With the *p* values of the *t* test all being below 0.05, the VIP value from the OPLS-DA model being ≥1.0 and FC > 2 or <0.5, among day 7 hemolymph metabolites, a total of 86, 87, and 126 components were assigned biomarker potential for pollen nutrition in pear vs. apricot, pear vs. rape, apricot vs. rape, respectively. Furthermore, among day 14 metabolites, a total of 56, 68, and 47 components were assigned biomarker potential for pollen nutrition in pear vs. apricot, pear vs. rape, and apricot vs. rape, respectively.

**Figure 4:**
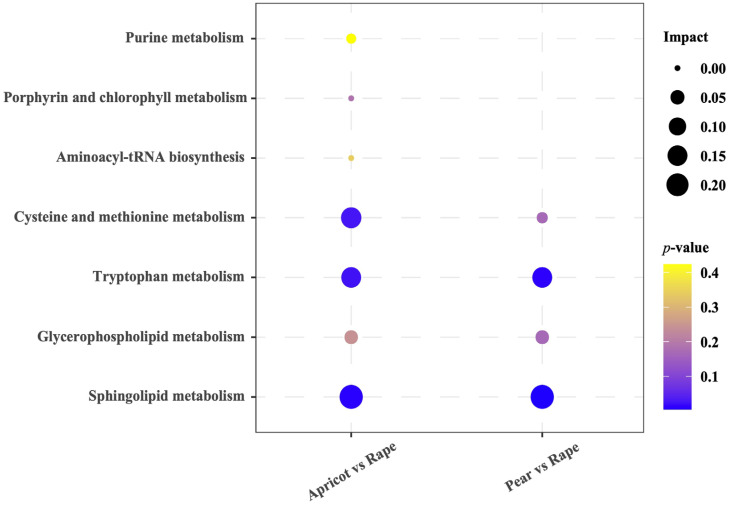
Pathway enrichment analysis of significantly altered metabolites on day 7. The larger the bubble is, the greater the influencing factor, the darker the color, the smaller the *p* value, and the more significant the degree of enrichment.

Our results showed that the metabolites of the day 7 (pear vs. apricot) and day 14 groups (pear vs. apricot, pear vs. rape, apricot vs. rape) could not be analyzed for meaningful pathway enrichment. In the day 7 groups, the 10 altered metabolites (sphinganine, phytosphingosine, 5-hydroxykynurenamine, 5-hydroxy-L-tryptophan 5′-methylthioadenosine, L-methionine, biliverdin, 1-acyl-sn-glycero-3-phosphocholine, L-methionine, urate) detected in apricot vs. rape were mapped to 7 KEGG metabolic pathways. Notably, the metabolic pathways “sphingolipid metabolism (*p* = 0.0091)”, “tryptophan metabolism (*p* = 0.0245)”, and “cysteine and methionine metabolism (*p* = 0.0277)” were significantly affected. In addition, the six altered metabolites detected in pear vs. rape were mapped to4 KEGG metabolic pathways, and the metabolic pathways “sphingolipid metabolism (*p* = 0.0039)” and “tryptophan metabolism (*p* = 0.0108)” were significantly affected (Figure 4).

### 3.4. Correlation Analysis of Amino Acids and Differential Metabolites

**Figure 5:**
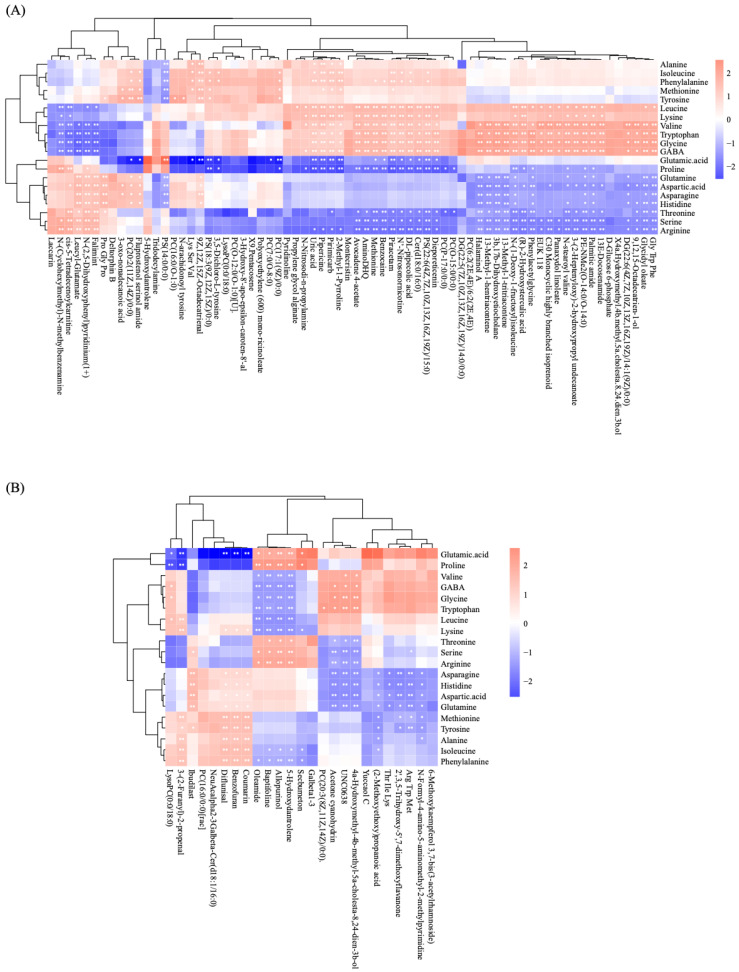
Correlation heatmap showing the relationship between the amino acid content and differentially abundant metabolites in hemolymph (*: 0.01< *p* < 0.05, and **: *p* < 0.01). The metabolites were identified in the METLIN database with a score greater than 80 and mass error less than 5 ppm (i.e., 0.0005%). (**A**) Hemolymph metabolites after feeding with different pollens for 7 days. (**B**) Hemolymph metabolites after feeding with different pollens for 14 days.

Spearman’s correlation analysis was performed between the differentially abundant metabolites and amino acid content of the three bee pollens. Our results showed that the number of metabolites of nurse-age bees on day 14 was significantly lower than that on day 7. Among the day 7 differentially abundant metabolites, our results show a positive correlation between the most essential amino acids (leucine, isoleucine, and valine) and a number of differentially abundant metabolites, including Lys-Ser-Val (*p* < 0.01) and piracetam (*p* < 0.01) (Figure 5A). Among the essential amino acids, the correlations of lysine and tryptophan with differentially abundant metabolites and the most essential amino acids showed the same trend. Among the day 14 differentially abundant metabolites, correlation analysis revealed a positive correlation of leucine and valine with UNC0638 (*p* < 0.01), 4-alpha-hydroxymethyl-4beta-methyl-5alpha-cholesta-8,24-dien-3beta-ol (*p* < 0.01) and 4-methoxybenzyl-O-(2-sulfoglucoside) (*p* < 0.01) (Figure 5B).

## 4. Discussion

Pollen is the only source of protein for bee colonies and is essential for brood rearing and the glandular development of young worker bees [8]. The nutrients in pollen vary greatly among plant species [5]. Our study showed that pear pollen had high nutritional value, wherein the levels of five essential amino acids (isoleucine, phenylalanine, lysine, methionine, and histidine) were the highest. We found differences in hemolymph metabolites in bees feeding on three bee pollens, wherein the number of metabolites on day 14 was significantly lower than that on day 7. We found that in early adulthood (the first 7 days), pollen affected related amino acid and lipid metabolism in honey bees. This study provides a deeper understanding of the effects of these three types of bee pollen on adult bee growth and development.

Different pollens have different levels of protein components [30]. Our results showed that GABA was the only nonprotein amino acid among the amino acids measured from the three bee pollens, which was also documented in De Groot’s (1953) study [12]. GABA is an important neurotransmitter in insect nervous systems, and studies have found that it significantly enhances the collection performance of honey bees [31,32]. The differences in the nutrient content of these three pollens also affect the foraging behavior of bees. A study revealed that honey bees show a preference for pollen rich in most essential amino acids [9]. It is known that optimal protein nutrition should provide a balanced content of essential amino acids that are required by honey bees [12,33]. Determination of the amino acid content in different pollens can be used to evaluate the nutritional quality of pollen [9]. Our results suggested that the levels of five essential amino acids (isoleucine, phenylalanine, lysine, methionine, and histidine) were the highest in pear pollen, and four of them were significantly higher than those in the other two kinds of pollen (*p* < 0.05). These results indicate that pear pollen had the highest nutritional value among the three pollens, followed by rape and apricot pollen, at the amino acid level, which is consistent with the notion that pollens with a high proportion of essential amino acids are of greater nutritional value than pollens with a low proportion of essential amino acids [7]. However, honey bees showed a significant preference for apricot pollen over pear pollen [17]. Our results also showed that proline was most abundant in apricot pollen (9777.50 ± 78.11 mg/kg). Interestingly, proline is involved in many physiological processes, and honey bees prefer nectar rich in proline [34,35,36,37], which seems to be a likely explanation for the bees’ foraging preference for proline-rich apricot pollen. Furthermore, because pear and field apricot pollens may differ in other properties, the effects of these on the preference of honey bees cannot be ruled out, such as differences in pollen color and odor among pear and apricot pollens.

Metabolites in the hemolymph may provide clues that connect pollen nutrition with worker metabolism. Our results showed that the number of metabolites on day 14 (615) was significantly lower than that on day 7 (1466), indicating that age itself already had a clear metabolic effect. It is possible that pollen is consumed primarily during the first 5 days of a honey bee’s life [18], and is later mainly used to obtain a single nutrient—sucrose. Furthermore, amino acids and phospholipids were the main differentially abundant metabolites in the hemolymph of bees during pollen feeding for 7 days, which provided new insights into the metabolic pathways of the differentially abundant metabolites.

The detection of hemolymph metabolites highlights the difference in the metabolism of a single type of pollen among honey bees, suggesting that pollen availability changes bee metabolic states. Through the differential metabolite maps constructed based on metabolite databases such as KEGG, we identified “sphingolipid metabolism”, “tryptophan metabolism”, and “cysteine and methionine metabolism” as three metabolic pathways of interest, as all of them exhibited lower *p* values and greater pathway impact (apricot vs. rape) (Figure 4). Sphingolipids are ubiquitous constituents of membrane lipids in eukaryotes, and sphingolipid metabolites have been revealed to modulate various cellular events, including proliferation, differentiation, and apoptosis [38,39]. Tryptophan metabolism has been studied extensively in insects, primarily because it is the initial precursor in the formation of ommochromes that are responsible for the color of wings and eyes in insects [40,41]. In addition, tryptophan plays regulatory roles in the growth, development, olfactory learning, memory abilities, and some physiological and biochemical properties of worker bees [42,43]. A proper amount of dietary tryptophan was found to significantly increase the concentrations of tryptophan and kynurenine and could stimulate hypopharyngeal gland development [43,44]. Kynurenines is an endogenous metabolite of tryptophan, and kynurenine metabolism can cause hyperactivity in honey bees [44,45]. Methionine is an essential amino acid and a precursor for cysteine and other metabolic intermediates, and it affects starvation tolerance and lifespan in *Drosophila* [46,47]. In addition, we also identified the metabolic pathways “sphingolipid metabolism” and “tryptophan metabolism” as being affected in the apricot vs. rape group (Figure 4). Our results suggest that the methionine content did not differ significantly between apricot and rape pollens (*p* > 0.05) (Figure 1). However, the differentially abundant metabolites in the apricot vs. rape group significantly affected the “cysteine and methionine metabolism” pathway (*p* < 0.05) (Figure 4), which may be due to the metabolic pathway changes caused by other substances in pollen. Therefore, differences in pollen nutritional qualities can affect not only related amino acid metabolism pathways but also related lipid metabolism in honey bees. Furthermore, our results also showed that the metabolites of the day 7 (pear vs. apricot) and day 14 (pear vs. apricot, pear vs. rape, apricot vs. rape) groups could not be analyzed for meaningful pathway enrichment. This result may be due to the close relationship between pears and apricots, and the differences in the metabolites of pollen may not be due to differences in metabolic pathways. On the other hand, some metabolites were not identified in the database, which may be because these compounds were not annotated in the database.

Bee pollens with different amino acid levels were metabolized in different ways by bees. Our results showed significant correlations between different amino acid species and differentially abundant metabolites in the hemolymph. The most essential amino acids (leucine, isoleucine, and valine) all showed a significant positive correlation with piracetam on day 7. Piracetam is a cyclic derivative of the neurotransmitter GABA, which has cognitive, neural, and vascular functions, but the mechanism of action remains unknown [48]. Furthermore, piracetam has been shown to exert a neuroprotective effect by decreasing the level of lipofuscin, an indicator of neuronal membrane damage [49]. These results suggest that pollen affects neurodevelopment in early adulthood. In addition, pollen in early adulthood continues to shape amino acid levels in the brain with age, which may affect neural circuitry and behavior over time [50]. However, many nonessential amino acids (e.g., aspartic acid, glutamic acid, cysteine, glycine, alanine, tyrosine, and proline) exhibited a positive or negative correlation with the differentially abundant metabolites in the hemolymph. Proline, for example, was the most abundant amino acid in these three pollens in our study. Proline is present in the hemolymph at higher concentrations than other amino acids, and it is used to a much greater extent in oxidative metabolism under nonflight conditions [51]. Moreover, we only evaluated the differences in amino acids, the main substances in these three pollen species, providing a theoretical basis for exploring the effects of other substances in pollen on bee metabolism.

## 5. Conclusions

In summary, our observations demonstrated that pear pollen had more species of essential amino acids and had higher nutritional value than the other two bee pollen types, and the difference in nutritional value among bee pollens effectively altered the metabolic state of adult honey bees. These results provide new insights into the responses of bees to different floral resources. Honey bees showed a significant preference for rape and apricot pollen over pear pollen, which was not determined by the nutritional value of the bee pollen. Interestingly, we found that the different bee pollens played important roles associated with amino acid and lipid metabolism pathways in honey bees during early adulthood. The metabolites and pathways detected in this study may provide valuable information for understanding the molecular basis of the complex interactions between bee pollen and bees.

## Data Availability

The data presented in this study are available on request from the corresponding author.
